# *pyDiSCaMB*: enabling the use of multipolar scattering factors in *Phenix*

**DOI:** 10.1107/S1600576726000828

**Published:** 2026-03-20

**Authors:** Viljar J. Femoen, Laura Pacoste, Michał Leszek Chodkiewicz, Pavel V. Afonine, Billy K. Poon, Marta Kulik, Łukasz Golon, Nigel W. Moriarty, Paul D. Adams, Gerhard Hofer, Paulina Maria Dominiak, Dorothee Liebschner, Xiaodong Zou

**Affiliations:** a Department of Chemistry University of Stockholm Stockholm Sweden; b Biological and Chemical Research Center, Faculty of Chemistry University of Warsaw Warsaw Poland; c Molecular Biophysics and Integrated Bioimaging Division Lawrence Berkeley National Laboratory Berkeley CA USA; d Department of Bioengineering, University of California, Berkeley, CA 94720, USA; Shiv Nadar Institution of Eminence, India

**Keywords:** transferable aspherical atom model, macromolecular refinement, *Phenix*, cryoEM, quantum crystallography, MATTS data bank, *cctbx*

## Abstract

Multipolar scattering models, such as the transferable aspherical atom model, account for atomic chemical interactions and provide a more accurate representation of experimental data. However, the simpler independent atom model (IAM), which assumes non-interacting atoms, is the only model available in the most widely used macromolecular refinement programs. This is primarily because IAM offers a hard-to-beat combination of computational efficiency and modelling power at typical macromolecular resolutions. By contrast, more accurate multipolar modelling has historically been limited due to its computational cost and the absence of an interface between software capable of calculating structure factors and gradients based on multipolar models and software designed for macromolecular refinement. This work introduces *pyDiSCaMB*, a Python software package designed to integrate between the computational crystallography toolbox (*cctbx*) and the quantum crystallography library *DiSCaMB* (*Densities in Structural Chemistry and Molecular Biology*), thus enabling multipolar scattering models in *Phenix*’s toolkit. The implementation, features and capabilities of *pyDiSCaMB* are presented, the runtimes for the calculation of structure factor and target gradients with respect to atomic parameters are explored, and Fourier images of electrostatic potential, electron density and deformation maps are computed as illustrative examples. The *pyDiSCaMB* library will make multipolar modelling widely available to the structural biology community, potentially transforming refinement and model-building for both crystallography and cryogenic electron microscopy (cryoEM).

## Abstract 2

*pyDiSCaMB* provides an interface between *cctbx* and *DiSCaMB*. The former drives *Phenix* and the latter provides multipolar parameterization of scattering, thus enabling multipolar modelling in *Phenix*.

## 1. Introduction

Crystallography and cryogenic electron microscopy (cryoEM) calculations – ranging from basic map interpretation and *R*-factor calculations to complex tasks like refinement and model building – typically rely on the independent atom model (IAM) to model atomic scattering. By modelling each atom as an isolated non-interacting sphere and disregarding different chemical environments, IAM offers an effective compromise between computational efficiency and accuracy across most data resolutions. In practice, IAM scattering factors are most commonly represented by a sum of Gaussians: four Gaussians plus a constant (Maslen *et al.*, 2006 ▸), five Gaussians (Waasmaier & Kirfel, 1995 ▸) or a dynamically defined number of Gaussians to approximate tabulated scattering factors (Grosse-Kunstleve *et al.*, 2004 ▸). In all of the aforementioned cases, the parameters of the Gaussians are tabulated per element, *i.e.* no matter what the chemical context or local environ­ment, all atoms of the same element are modelled with the same parameters. We note that there are tabulated values for ions but they all assume formal integer charges.

Despite its usefulness, IAM has well known limitations. In X-ray crystallography, the IAM approach breaks down at ultra-high resolutions (better than ∼0.7 Å), where the data begin to reveal electron-density features incompatible with the model’s assumption that the atoms are spherical and chemically non-interacting (Guillot *et al.*, 2008 ▸; Afonine *et al.*, 2004 ▸; Jelsch *et al.*, 2000 ▸). Even though additional spherical scattering sites located on the covalent bonds can be modelled with interatomic scatterers to complement IAM (Afonine *et al.*, 2007 ▸), this approach does not account for the densities corresponding to electron lone pairs in areas with dipole–dipole or hydrogen-bond interactions. Thus, the aspherical character of the electron density cannot be accurately modelled with spherical atoms (Kulik & Dominiak, 2022 ▸). In electron crystallography and cryoEM, IAM proves inadequate more fundamentally, across all resolutions, due to the intrinsic nature of electrostatic potential maps, which cannot be accurately represented by atom-centred spherical densities (Kulik *et al.*, 2022 ▸; Kumar *et al.*, 2019 ▸; Marques *et al.*, 2019 ▸).

The multipolar model formalized by Hansen & Coppens (1978 ▸) presents a more accurate alternative for both crystallography and cryoEM. In this formalism, the electron density is modelled with a fixed spherical density from tabulated data, a spherical valence shell allowing for charge transfer between atoms, and a multipole aspherical deformation term expressed by spherical harmonics and radial functions. Despite this model being able to describe atomic densities more realistically, its use has been largely restricted to the small-molecule community due to its computational complexity, increased parameter space and prohibitive runtime scaling for larger structures. Indeed, the number of refinable parameters for an atom (described with anisotropic *B* factors) increases substantially for the multipolar model, *e.g.* from 10 up to 37 per atom when including up to hexadecapoles. Thus, the data need to be of ultra-high quality and resolution (*d* ≤ 0.5 Å) in order to refine the multipolar parameters against the experimental data (Dittrich *et al.*, 2009 ▸). Such data are rarely obtainable for proteins, with only two examples known to date in the Protein Data Bank (PDB, https://www.rcsb.org/; Berman *et al.*, 2000 ▸): 3nir (Schmidt *et al.*, 2011 ▸) and 5d8v (Hirano *et al.*, 2016 ▸). With slightly worse resolution, full or partial multipolar refinement has also been attempted, but only under strong constraints and usually limited to well ordered subsets of atoms, such as reported by Jelsch *et al.* (1998 ▸), Housset *et al.* (2000 ▸) and Guillot *et al.* (2008 ▸).

As a practical alternative, the requirement for such data and the computational cost can be alleviated by using tabulated values of multipolar parameters for different local environments of atoms instead of refining them. This approach is called the transferable aspherical atom model (TAAM), where atoms are assigned to specific atom types on the basis of their chemical environment and receive the corresponding tabulated scattering properties. For example, a Cα carbon atom in an amino acid residue will be assigned a different atom type from a carbon atom with *sp*^3^ geometry in a methyl (CH_3_) group. The assignment of the atom type is therefore a complex operation that needs to be performed in order to assign multipolar parameters successfully. The transferable atom types and corresponding multipolar parameters are tabulated in different libraries, such as the UBDB (Dominiak *et al.*, 2007 ▸), later superseded by MATTS (Jha *et al.*, 2022 ▸; Rybicka *et al.*, 2022 ▸), the ELMAM2 database (Domagała *et al.*, 2012 ▸) and the Invariom database (Dittrich *et al.*, 2013 ▸). The libraries differ in the origin of the multipolar parameters: ELMAM2 is based on parameters derived from multipolar refinements against high-resolution diffraction data, Invariom on geometry-optimized theoretical model compounds and MATTS/UBDB on averaged parameters derived from theoretical calculations on experimentally determined geometries. Using fixed precomputed multipolar parameter values for tabulated local atomic environments reduces the number of refined parameters down to the same number as with IAM, while retaining the increased accuracy of the multipolar model. TAAM is available in some structure refinement packages for small molecules, *e.g. OLEX2* (Dolomanov *et al.*, 2009 ▸; Jha *et al.*, 2020 ▸, 2022 ▸), *JANA2020* (Petříček *et al.*, 2023 ▸, 2014 ▸) and *XD2024* (Volkov *et al.*, 2024 ▸). *MoPro* (Guillot *et al.*, 2001 ▸) supports refinement of both small molecules and protein structures and was a pioneer in enabling TAAM refinement of macromolecules (Jha *et al.*, 2021 ▸; Jelsch *et al.*, 2005 ▸), but it currently does not implement several automation and workflow features that are standard in widely adopted macromolecular crystallography software packages, and it lacks support for refinement against cryoEM data.

*DiSCaMB* (*Densities in Structural Chemistry and Molecular Biology*; Chodkiewicz *et al.*, 2018 ▸) is a standalone C++ library that provides an implementation of aspherical models of atomic form factors, including TAAM-based calculations based on the MATTS database (Jha *et al.*, 2022 ▸; Rybicka *et al.*, 2022 ▸). Its functionality includes automated atom type assignment based on local chemical environment and subsequent assignment of multipolar parameters from transferable atom databases. Using these parameters, *DiSCaMB* enables the computation of TAAM-based X-ray and electron structure factors, as well as their derivatives.

Here, we introduce *pyDiSCaMB*, a Python-based interface to the existing *DiSCaMB* library. The *pyDiSCaMB* interface enables the use of the multipolar model within the computational crystallography toolbox *cctbx* (Grosse-Kunstleve *et al.*, 2002 ▸) and *Phenix* (Liebschner *et al.*, 2019 ▸; Adams *et al.*, 2010 ▸, 2002 ▸).

Although *DiSCaMB* and *cctbx*/*Phenix* each use C++, their internal object models differ. The purpose of *pyDiSCaMB* is to provide an interface that translates between the corresponding data structures and functionalities used in these frameworks.

This article describes the implementation of *pyDiSCaMB*, summarizes its features and capabilities, explores runtimes for structure factor and gradient calculations, and illustrates its use. Integration with *Phenix* will enable the broader structural biology community to access the enhanced accuracy of multipolar modelling, potentially transforming atomic model refinement for both crystallography and cryoEM.

## 2. Features and capabilities

**Figure 1:**
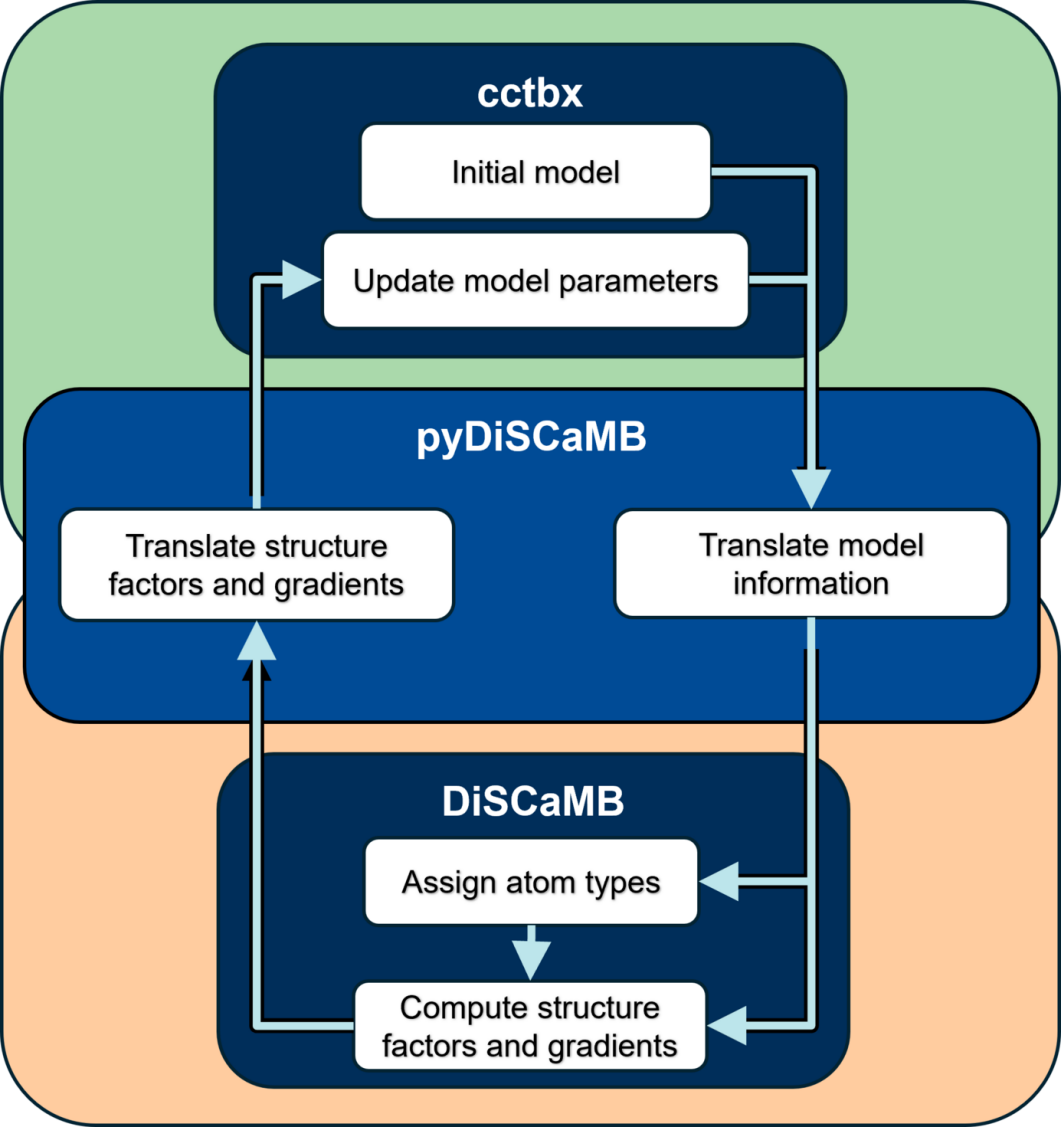
Flowchart illustrating how *pyDiSCaMB* enables atomic model refinement in *Phenix* by serving as an interface layer between *cctbx* and *DiSCaMB*. *pyDiSCaMB* takes model information from *cctbx* and translates it into a format suitable for *DiSCaMB*. The model information is used by *DiSCaMB* to perform atom type assignment, and it then computes structure factors and necessary derivatives with respect to atomic model parameters using either IAM or TAAM. These calculated values are returned to *cctbx* through *pyDiSCaMB* and used for refinement of the atomic model.

*pyDiSCaMB* provides an interface to *DiSCaMB*’s structure factor and gradient calculators via the DiscambWrapper class. These calculators compute structure factors and their gradients, which can be used for model refinement (Fig. 1 ▸). All *DiSCaMB* calculators are available through an initialization interface that uses input provided in JSON format. These input parameters can be conveniently specified as keyword arguments to the DiscambWrapper.

For convenience, two calculation modes are available, TAAM and IAM. For TAAM, the MATTS data bank (Jha *et al.*, 2022 ▸; Rybicka *et al.*, 2022 ▸) provides parameterization for X-ray scattering, with the option of converting to electron scattering using the Mott–Bethe formula (Massey, 1956 ▸). When TAAM mode is enabled, *DiSCaMB* accesses the MATTS data bank, which provides a dictionary defining the multipolar type of each atom and its associated local coordinate system. Atom types are then assigned by *DiSCaMB* on the basis of this information.

The atom type assignment in *DiSCaMB* can result in three possible representations for each atom. When parameters from the MATTS data bank are available, the atoms are assigned a complete multipolar (TAAM) representation. If no multipolar parameters are available, *DiSCaMB* assigns a spherical Hansen–Coppens model, using only the core and spherical valence terms of the model based on neutral and isolated atoms. In practice, these are alternative IAM representations based on Slater-type radial functions from Clementi–Roetti calculations (Clementi & Roetti, 1974 ▸), which are used in *DiSCaMB* for atoms up to krypton (*Z* ≤ 36). If neither of these assignments is possible, the atoms are modelled with a Gaussian parameterization of the IAM for neutral atoms, as in conventional refinement packages.

For accurate atom type assignment there are a few things to consider. The current typing algorithm uses only atomic coordinates and element types from each atom, together with predefined covalent-bond radii and tolerance thresholds, to identify that atom’s local environment. This is defined by its first neighbours (directly bonded atoms), second neighbours (atoms two bonds away) and sometimes neighbours farther away. *DiSCaMB* accordingly determines bonding connectivity and topological features such as planarity, ring membership and local geometry. Therefore, hydrogen atoms must be present in the model, as many types rely on bonded hydrogen atoms. Alternative conformations or clashes can result in overlapping atomic positions that interfere with the accurate determination of bonding connectivity. This can lead to an incorrect interpretation of the bonding environment and assignment of IAM to such atoms.

When IAM mode is enabled, *DiSCaMB* uses a Gaussian neutral IAM representation for all atoms. Several tables of Gaussian parameters are available for scattering factors. This includes Waasmaier & Kirfel’s (1995 ▸) parameterization for X-rays, the X-ray parameterization in *International Tables for Crystallography*, Vol. C (IT-C; Maslen *et al.*, 1992 ▸), and the corresponding parameterization for electron scattering in IT-C (Cowley *et al.*, 2006 ▸), which are provided for two scattering-vector ranges: up to 2 Å^−1^ and up to 6 Å^−1^, corresponding to the limits over which the fits are considered reliable.

## 3. Low-overhead implementation

*pyDiSCaMB* is written as a thin wrapper of *DiSCaMB*, mainly translating between *cctbx*’s structure object and *DiSCaMB*’s crystal class. To reduce the number of *DiSCaMB* internal classes and objects that are exposed to Python, the structural information is translated on the C++ side of *pyDiSCaMB*. This simplifies the user interface to a single class (DiscambWrapper) while retaining the option of multiple structure factor calculation classes by means of a parameter dictionary. The Python side of *pyDiSCaMB* handles the TAAM parameter data bank and provides helper functions to initialize a DiscambWrapper object from model files in CIF (for small molecules), mmCIF (for macromolecules) and PDB formats.

The task of *pyDiSCaMB* is to act as a translation layer between the refinement algorithm and *DiSCaMB*. The translation inevitably introduces some overhead. To determine its impact on the runtime, each function call was timed when calculating structure factor gradients from *cctbx*. As a test case, we used the PDB model 7der (Tanaka *et al.*, 2021 ▸) of lysozyme (2195 atoms, including hydrogen atoms at neutron *X*—H distances, were used in the calculations) at 1.03 Å resolution. As gradients are computed for each refinement cycle, this gives an estimate of the relative impact of the wrapper.

**Figure 2:**
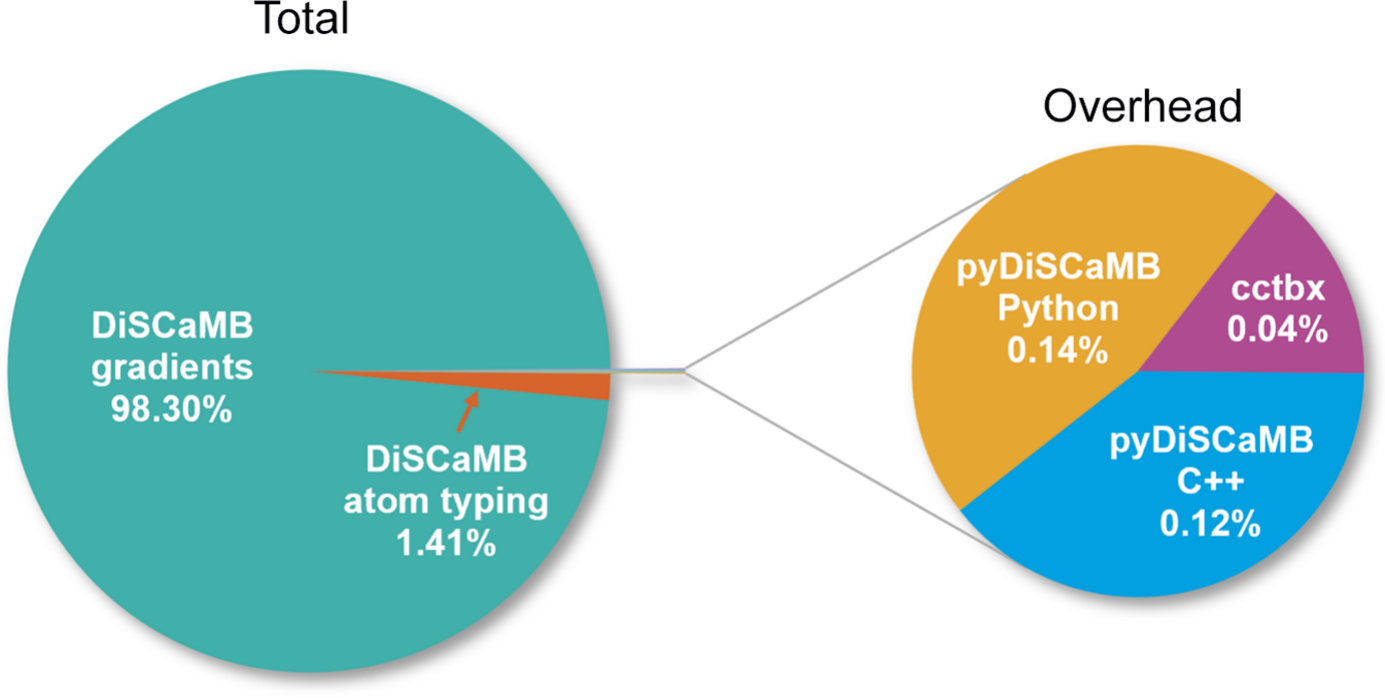
Distribution of runtime when computing TAAM target gradients from *cctbx* for PDB model 7der (1.03 Å resolution). The right-hand pie chart represents the 0.3% of runtime not spent by *DiSCaMB*.

Calls to C++ functions were measured using chrono: high_resolution_clock and Python calls with time.perf_counter. Fig. 2 ▸ shows the distribution of runtime. The test indicates that less than 0.3% of runtime is spent by the *pyDiSCaMB* wrapper for a typical use case, while 99.7% is spent on *DiSCaMB* computations. The overhead therefore represents only a fraction of runtime. This aligns well with the aim of keeping overhead low. We also note that the atom typing procedure takes up less than 1.5% of runtime. The majority of time (98.3%) is therefore spent on the actual calculation of gradients.

For structure refinement, the typical approach is to perform minimization of a target function, *e.g.* the sum of squared differences between observed and computed structure factor amplitudes (least squares), which is used in small-molecule crystallography, or versions of maximum-likelihood-based functions primarily used in macromolecular crystallography (Lunin & Urzhumtsev, 1984 ▸; Read, 1986 ▸, 1990 ▸; Bricogne & Irwin, 1996 ▸; Pannu & Read, 1996 ▸; Murshudov *et al.*, 1997 ▸; Adams *et al.*, 1997 ▸; Tickle *et al.*, 1998 ▸; Pannu *et al.*, 1998 ▸). For each iteration, the refined parameters are nudged in the direction opposite to the gradient of the target function with respect to the parameters, thereby reducing the value of the target function. *DiSCaMB* has two options to compute gradients: structure factor gradients with respect to atomic parameters, or target gradients with respect to atomic parameters. Calculating target gradients requires target function gradients with respect to structure factors as input from *cctbx*, which already provides these values. However, this additional input communication comes with the benefit of a greatly reduced output communication. If all structure factor gradients with respect to atomic parameters were to be transferred to *cctbx* for accumulation into the final target gradients, it would require memory proportional to both the number of parameters and the number of reflections. This is easily of the order of hundreds of gigabytes, whereas providing *DiSCaMB* with target gradients with respect to structure factors results in outputs proportional only to the number of parameters, reducing the typical size to megabytes.

The package uses *scikit-build* (Fillion-Robin *et al.*, 2018 ▸) as a build backend, which integrates with *DiSCaMB*’s existing *CMake* (kitware Inc.; https://cmake.org/) build system. Python bindings for the C++ code are made using *pybind11* (Jakob *et al.*, 2017 ▸), simplifying the process of interfacing between the languages. *pybind11* was used in favour of *Boost.Python* (Abrahams & Grosse-Kunstleve, 2003 ▸), which is used in *cctbx*, as the rest of the *Boost* library is not needed and *pybind11* produces smaller binaries (Lyskov, 2016 ▸). Tests are performed with *pytest* (Krekel *et al.*, 2004 ▸). They include comparisons between neutral IAM structure factors computed with *cctbx* and *DiSCaMB*, assertion of correctly recognized input parameters and expected outputs, and checking of TAAM atom typing. The test suite is automatically run using *GitHub Actions* for all changes to the code and runs the tests for multiple Python versions, operating systems and C++ compilers, ensuring the code works as intended in a variety of setups. Specifically, automatic testing is performed for Python Versions 3.9 to 3.13, using *GCC* 11.4 on Ubuntu 24.04, *MSVC* 19.44 on Windows Server 2022 and *Clang* 20.1.8 on macOS 14. Additional tests are performed daily with *cctbx*’s test suite.

## 4. Runtime comparisons

As the aspherical model is more complex than the IAM model, the computations are also more demanding, and as a consequence this affects runtime. *cctbx* uses a fast Fourier transform (FFT)-based algorithm for IAM (Ten Eyck, 1977 ▸; Sayre, 1951 ▸; Afonine & Urzhumtsev, 2004 ▸). Since in IAM atoms are treated as non-interacting Gaussian spheres, and since the Fourier transform maps Gaussians to Gaussians and – by the convolution theorem – maps convolutions to products (so products of Gaussians are Gaussians), FFT-based algorithms can compute structure factors and their gradients much faster than direct summation. These FFT-based approaches are not available for densities based on the Hansen–Coppens multipole model, since these are not modelled as non-interacting Gaussian spheres. As a result, structure factors for TAAM in *DiSCaMB* are calculated using the direct summation approach. This method scales with the product of the number of reflections and roughly the number of atoms in the unit cell. Additionally, assigning atom types scales linearly with the number of atoms, although in practice it constitutes a marginal fraction of runtime (Fig. 2 ▸) because this is performed only once.

To compare the runtimes of different structure factor calculators, we measured the runtimes for different structures and calculation methods. The calculators used were *cctbx*, using both FFT and direct summation IAM, and *DiSCaMB*, using IAM and TAAM. Five structures from the PDB, which cover a range of structure sizes (600–20000 atoms), resolutions (0.48–2.00 Å) and numbers of reflections (20000–160000 reflections), were tested: 3nir (crambin; Schmidt *et al.*, 2011 ▸), 7der (lysozyme; Tanaka *et al.*, 2021 ▸), 6g1t (cytosolic protein TraN; Kohler *et al.*, 2018 ▸), 6ger (β-amylase; Hofer *et al.*, 2019 ▸) and 6ipu (human nucleosome core particle containing 145 bp of DNA; Sharma *et al.*, 2019 ▸). Note that we are using the human readable codes established following Moriarty (2015 ▸).

To ensure consistent treatment of all atoms during the TAAM computations, each test model was prepared so that multipolar atom types could be assigned to all atoms. Water molecules, ions, ligands and alternative conformations were removed, incomplete C-termini in protein chains and 5′-termini in DNA chains were completed, and element labels were standardized to neutral form (as oxygen atoms on phosphate groups in DNA chains are sometimes given a negative O^−^ label). For all structures, hydrogen atoms were added or remodelled using *MolProbity* (Version 4.5.2; Williams *et al.*, 2018 ▸; Chen *et al.*, 2010 ▸; Davis *et al.*, 2004 ▸) at neutron *X*—H distances to ensure chemically reasonable geometry. Residues were renumbered so that each chain began with residue 1, as required by *MolProbity* to place hydrogen atoms on the N termini. After processing, all five structures had 100% of their atoms assigned a multipolar type.

For each of these structures, all four calculator setups were run five times each to verify that the results were stable and to ensure that the variability between runs was small. The computations were performed on a single core of an Intel Core i9-14900K CPU, using *cctbx-base* (Version 2025.10) and *pyDiSCaMB* (Version 0.4.1). Both structure factors and target gradients were computed for each run. As a refinement cycle needs both structure factors and target gradients, this will give an indication of the relative refinement runtimes for the different implementations. In addition, TAAM computations were repeated using a multi-thread system (MT; 16 cores/32 threads) under the same conditions to assess the achievable speed-up.

**Figure 3:**
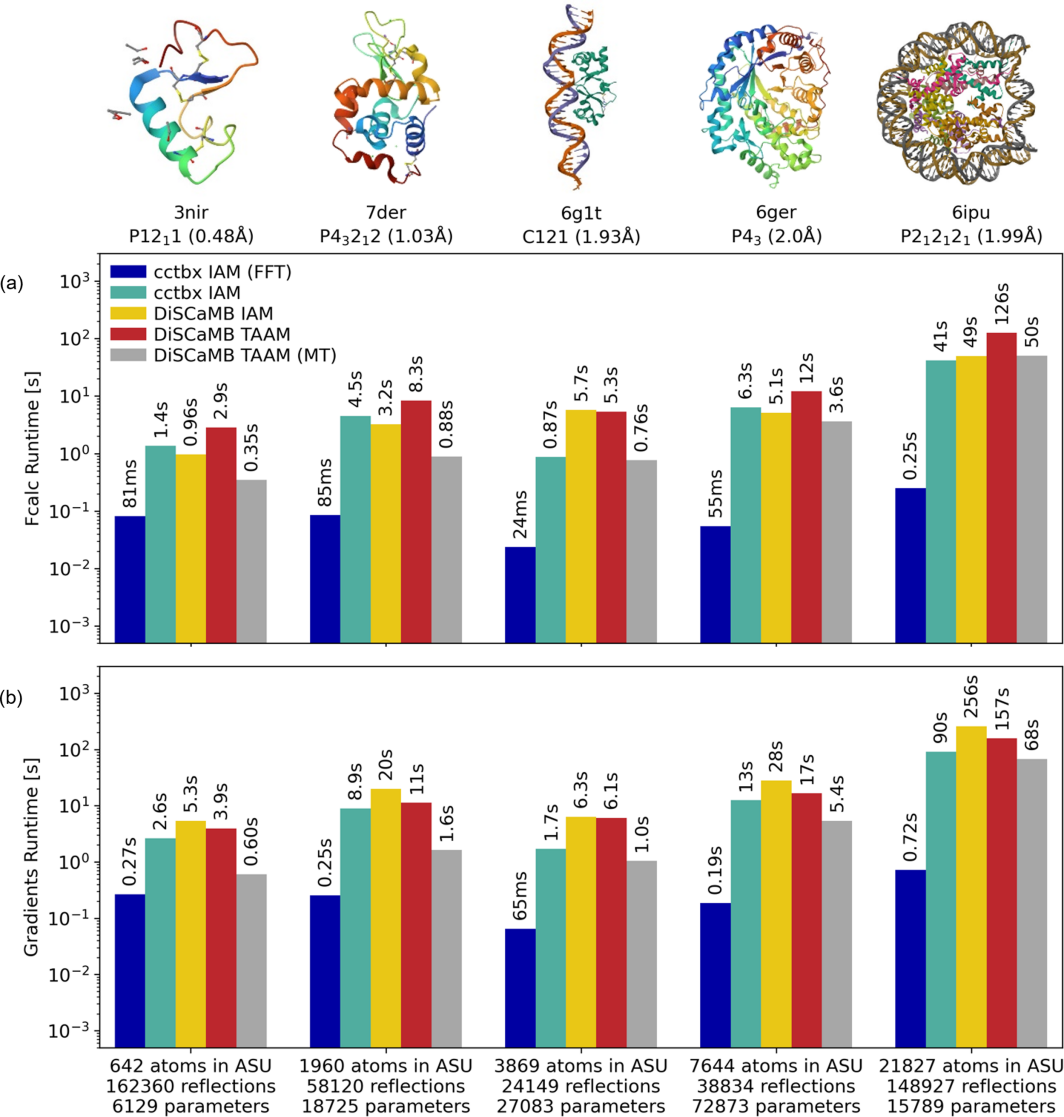
Mean runtimes (*n* = 5), presented on logarithmic time axes, for computing (*a*) structure factors and (*b*) target gradients with respect to atomic parameters (*x*, *y*, *z*, *B* factors and occupancies) for selected entries in the PDB. The computations are performed on a single CPU core, except for TAAM computations, which are performed on both a single CPU core and on a multi-thread system (MT) to demonstrate the achievable reduction in runtime. The space group and resolution are indicated at the top. The number of atoms (including hydrogen atoms) in the asymmetric unit (ASU), number of reflections and number of gradient parameters used are indicated at the bottom. Runtime is reported in seconds (s) or milliseconds (ms). Standard deviations were <3% and are not shown due to the logarithmic time axes (values are provided in Table S1 in the supporting information). Assembly images taken from the PDB.

The mean runtimes for computing structure factors and parameter gradients for the five entries are shown in Fig. 3 ▸. The standard deviations were <3% and are listed in Table S1 (supporting information). Current TAAM computations are around 10–100 times slower than *cctbx*’s FFT-based IAM algorithm. We note that for the larger structures (6ger and 6ipu), TAAM takes approximately the same time as direct summation IAM, especially in the case of gradients [Fig. 3 ▸(*b*)], demonstrating that the main computational limitation lies in the required use of the direct summation algorithm, rather than being intrinsic to the use of TAAM. Current efforts are focused on improving the performance of structure factor calculations using direct summation in *DiSCaMB* (Appendix *A*[App appa]).

Note that the benchmarks discussed here were performed on a single CPU core to provide a direct comparison of algorithmic performance. Since the *DiSCaMB* implementation supports parallel execution, substantial reductions in runtime could be achieved on MT systems. When the TAAM computations were performed on a 16-core/32-thread pro­ces­sor, the runtime decreased by approximately two to ten times depending on the number of atoms in the mol­ecule (Fig. 3 ▸).

## 5. Example Usage

**Figure 4:**
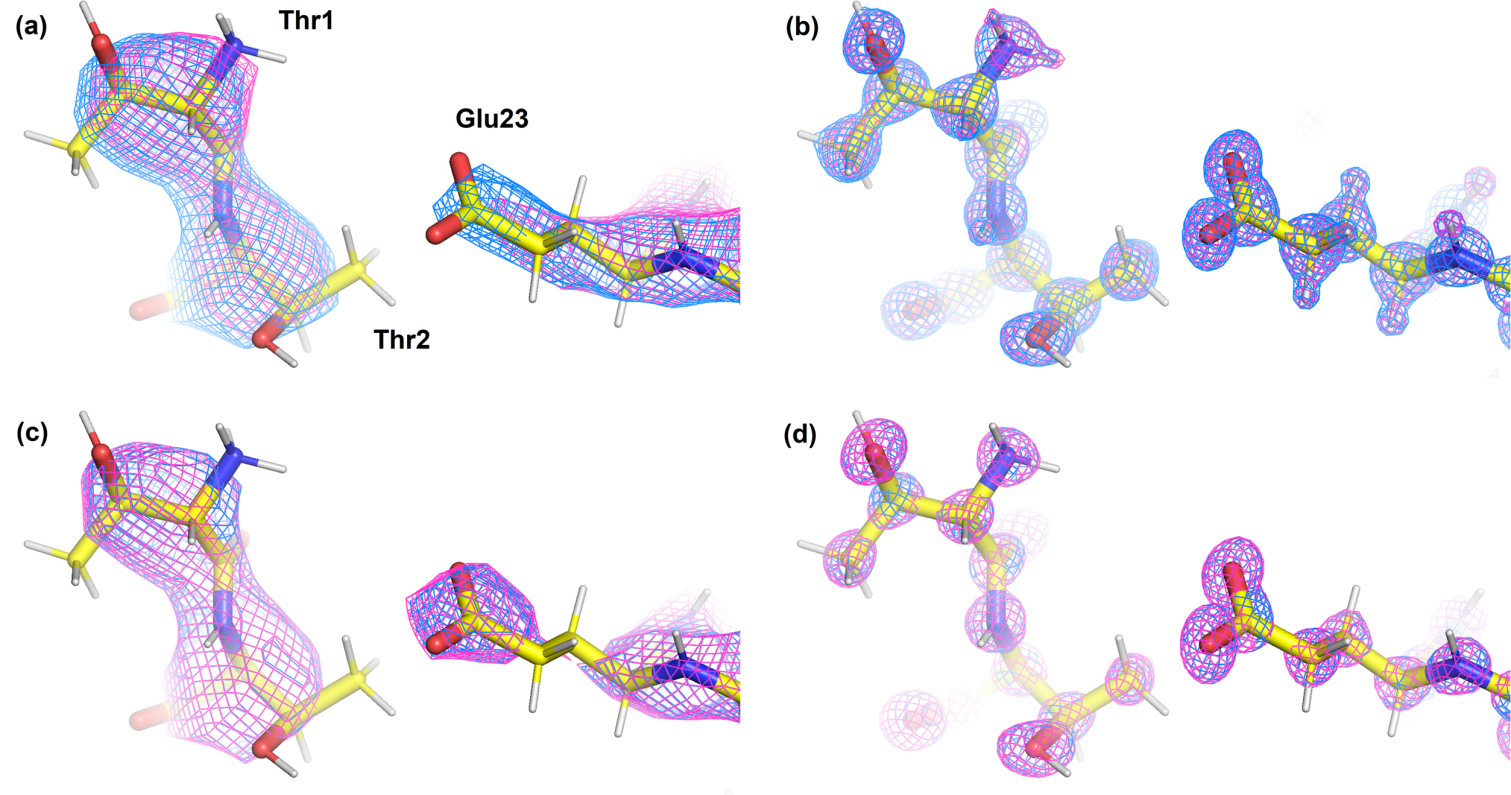
(Top row) Fourier electrostatic potential maps, calculated at (*a*) 4 Å resolution contoured at 0.195 Å^−2^ and (*b*) 0.7 Å resolution contoured at 0.425 Å^−2^, and (bottom row) Fourier electron-density maps, calculated at (*c*) 4 Å resolution contoured at 0.555 e Å^−3^ and (*d*) 0.7 Å resolution contoured at 1.465 e Å^−3^, for crambin (PDB ID 3nir) using TAAM (magenta) and neutral IAM (blue). Contour levels, displayed on an absolute scale, were set to the midpoint between the levels corresponding to 1.5 root-mean-square deviation in the IAM and TAAM maps. Note the difference in units between the Fourier electrostatic potential maps (Å^−2^) and electron density (e Å^−3^). (The unit of electron structure factors is the ångström, as defined in *International Tables for Crystallography* and adopted by *DiSCaMB* and *cctbx*. Consequently, the Fourier electrostatic potential map has units Å/Å^3^ = Å^−2^.)

The *pyDiSCaMB* library has the capability to generate Fourier images of electron-density and electrostatic potential maps for a given structural model via structure factor calculation. It provides a user-friendly implementation that enables the generation of Fourier electrostatic potential maps for macromolecules using TAAM with the MATTS data bank across a broad range of resolutions, without the need to combine many separate software packages. To demonstrate this, Fourier electrostatic potential and electron-density maps were generated with *pyDiSCaMB* using a Python script. Fig. 4 ▸(*a*) shows a Fourier electrostatic potential map calculated with TAAM for a protein structure (crambin, PDB ID 3nir). For comparison, the corresponding Fourier map was calculated with neutral IAM using data from IT-C for X-rays and electrons (Maslen *et al.*, 1992 ▸; Cowley *et al.*, 2006 ▸).

Even though the crambin structure was refined to 0.48 Å resolution, Fourier maps at lower resolutions, in particular close to 4 Å, are of particular interest due to the sensitivity of electron scattering to charged species at 4 Å resolution (Yonekura *et al.*, 2015 ▸; Marques *et al.*, 2019 ▸; Kulik & Dominiak, 2025 ▸), and clear differences between the TAAM and IAM electrostatic potential maps can be observed at the positions of charged residues. For example, the contour of the TAAM electrostatic potential map covers a larger volume around the positively charged terminal amino group of Thr1 and a smaller volume around the negatively charged Glu23 than the contour of the IAM map of crambin. Neutral residues, such as Thr2, exhibit similar map contours at the same contour level. At 0.7 Å resolution, minor differences close to the hydrogen atoms of Thr1 are still visible [Fig. 4 ▸(*b*)]. Fourier electron-density maps, characteristic for X-ray diffraction, do not reveal any sensitivity to charged residues, neither at 4 Å nor at 0.7 Å resolution [Figs. 4 ▸(*c*) and 4 ▸(*d*), respectively].

In order to capture and interpret small deviations between TAAM and IAM at high resolution, visual comparison of maps generated from the two models may be insufficient, as subtle differences may not be perceived. Fourier deformation maps, representing the difference between the aspherical TAAM density and the spherical IAM density, can also be computed using the *pyDiSCaMB* library. Such a map is calculated as a Fourier image computed from TAAM and IAM structure factor differences.

**Figure 5:**
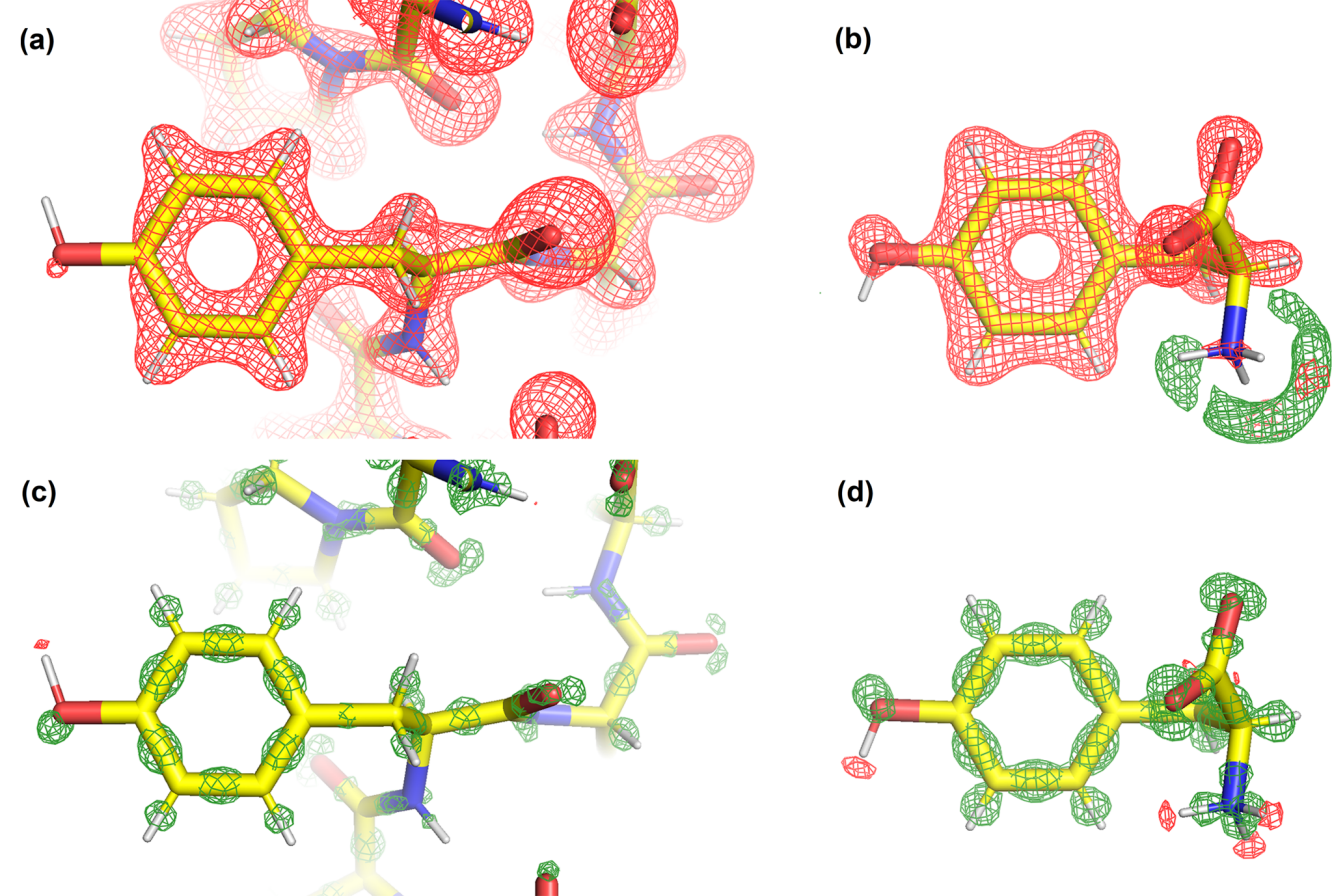
Fourier deformation maps of tyrosine calculated at 0.7 Å resolution for (*a*) and (*b*) electron diffraction (red contours at −0.060 Å^−2^, green contours at 0.060 Å^−2^) and (*c*) and (*d*) X-ray diffraction (red contours at −0.200 e Å^−3^, green contours at 0.200 e Å^−3^). Panels (*a*) and (*c*) show the Tyr44 residue in crambin (PDB ID 3nir). Panels (*b*) and (*d*) show the l-tyrosine crystal (CCDC No. 2479735). Contour levels were chosen for visual clarity and are shown on an absolute scale, corresponding approximately to the relative scale of 2 root-mean-square deviation (r.m.s.d.) for electron diffraction and 3 r.m.s.d. for X-ray diffraction deformation maps.

As an example, Fourier deformation maps were generated for tyrosine, both within crambin and in an l-tyrosine crystal [Cambridge Crystallographic Data Centre (CCDC) No. 2479735; Dominiak *et al.*, 2025 ▸], using *pyDiSCaMB* in a Python script (Fig. 5 ▸). This functionality has also been integrated into *Phenix* as the tool phenix.TAAM_minus_IAM, which includes an option to generate the maps with or without scaling between TAAM- and IAM-based structure factors. In these calculations, the maps were generated without scaling. The IAM structure factors were based on the Gaussian parameterizations from IT-C for X-ray (Maslen *et al.*, 1992 ▸) and electron scattering (Cowley *et al.*, 2006 ▸) valid to 6 Å^−1^, which are the default choices in *DiSCaMB* for the respective radiation type. The fragment of the Fourier electro­static potential deformation map shown in Fig. 5 ▸(*a*) calculated around Tyr44 in crambin features only negative values at the chosen contour level. The amino group of the l-tyrosine crystal in Fig. 5 ▸(*b*) is an example of a positively charged group generating strongly positive electrostatic potential, captured by TAAM. The positive peaks are absent in the electrostatic potential deformation map for the protein environment because the amino group forms a peptide bond and is therefore no longer positively charged. Figs. 5 ▸(*c*) and 5 ▸(*d*) show the electron-density deformation maps of tyrosine in crambin and the l-tyrosine crystal for X-ray scattering. Positive peaks in the middle of carbon–carbon covalent bonds and near oxygen atoms illustrate that IAM does not account for the electron density arising from bond formation and lone electron pairs, respectively. Negative peaks near hydrogen-atom positions reveal locations where IAM overestimates the contributions to the molecular electron density.

Although the electrostatic potential deformation maps shown here were calculated at the same resolution as the electron-density deformation maps, they do not exhibit such sharp peaks associated with lone-pair positions or covalent bonds as the electron-density deformation maps. This is because the electrostatic potential is a more spatially extended function than the electron density, spreading over a larger volume away from the nuclei. As a consequence, it appears more diffuse on the maps, with less-defined features for the covalent bonds and lone electron pairs. The electrostatic potential deformation maps represent the electrostatic potential function as it would be computed directly from electron-density deformation maps. Regions of electron excess are the source of negative electrostatic potential and regions of electron depletion are the source of positive potential. Contributions from nuclear charges are obviously not present on electrostatic potential deformation maps, as the nuclear charges in IAM and TAAM are exactly the same.

## 6. Discussion and outlook

This work represents an important step towards enabling more complex multipolar scattering models in the *Phenix* toolkit. The *pyDiSCaMB* library facilitates structure factor calculations based on TAAM for macromolecular atomic models. This functionality is already available in *cctbx* and can be integrated into data analysis scripts and pipelines.

Our results show that TAAM-based structure factor and target gradient calculations have comparable runtimes to existing direct summation IAM implementations, particularly for larger structures. While longer runtimes than FFT-based IAM are expected, this primarily reflects the use of the direct summation approach, rather than being a limitation of using TAAM in structure factor and gradient calculations. We note that no TAAM refinements are presented in this work. A comprehensive evaluation of refinement performance is planned as a follow-up study.

The described methods for structure factor and gradient calculations in *cctbx* and/or *Phenix* currently require installation of *pyDiSCaMB*, *DiSCaMB* and MATTS directly from the source code (described in Appendix *B*[App appb]). The methods can be applied to any protein or RNA/DNA model, including organic ligands, but there are some limitations that concern the atom type assignment procedure, *i.e.* depending on the content of the structural model, a certain proportion of atoms will not be assigned with the multipolar model. For example, this applies to atoms in multiple conformations or in fragments with very bad geometry and/or clashes (work to support these is ongoing), water molecules (typically the positions of H atoms needed to get a multipolar representation are unknown), incomplete entities (*e.g.* residues with missing atoms, which prevent the formulation of the local coordinate system for the bound existing atoms), and atoms other than C, H, N, O, S, P, F, Cl, Br and their closest neighbours (work to support other elements like B or I, and some of the specific metals and their coordination, is ongoing). These atoms will then be considered spherical, making it possible to perform downstream calculations. It is therefore recommended that prospective users take note of the percentage of atoms assigned as multipolar.

The current implementation represents an important step towards making TAAM refinement for macromolecules widely available, but further work is needed to reduce runtime and enable its routine use for large macromolecules as well. Since the *DiSCaMB* library already supports parallel execution, substantial speed-up can be achieved on multi-core systems and future developments may also exploit *DiSCaMB*’s GPU computing capabilities. Ongoing efforts will focus on improving the performance of the direct summation algorithm to make TAAM calculations faster and more widely applicable in practice.

Developments in *DiSCaMB* are intended to be integrated into *pyDiSCaMB* as they become available. This includes ongoing work to improve atom type assignment towards a dictionary-style method based on atom and residue identity, which will also facilitate assignment for alternative conformations and improper geometry. In addition, current developments aim to use crystallographic symmetry to reduce runtime. Continued development of *pyDiSCaMB* will aim to expose more of *DiSCaMB*’s extensive features, such as Hirshfeld atom refinement (Jayatilaka & Dittrich, 2008 ▸; Capelli *et al.*, 2014 ▸), which derives atomic scattering factors using quantum mechanics methods.

TAAM refinement for macromolecules is expected to have a significant impact on the refinement of a wide range of structures, improving the accuracy of detailed features such as *B* factors and the visualization of electron-density distribution or hydrogen-atom positions in the case of atomic resolution data. In particular, for refinement against electron diffraction and cryoEM data, TAAM is anticipated to provide a more accurate representation of the electrostatic potential map, even when the data are limited to low resolution (Fig. 4 ▸). For small molecules, the use of TAAM scattering factors has already been shown to reduce residual map noise, lower the *R* values, improve the accuracy of hydrogen-atom positions and provide more physically meaningful atomic displacement parameters (Gruza *et al.*, 2020 ▸; Olech *et al.*, 2024 ▸; Pacoste *et al.*, 2024 ▸). Extending the availability of TAAM to the refinement of macromolecules is expected to enhance the reliability of electrostatic sensitive features such as protonation states and characterization of bonding features. In addition, this approach may provide opportunities to probe valence states at metal sites using electron diffraction, offering a possible route to studying redox chemistry and charge redistribution in metalloenzymes.

## 7. Conclusions

The development of *pyDiSCaMB* provides a Python interface between *cctbx* and the quantum crystallography library *DiSCaMB*, enabling the use of multipolar scattering models in *Phenix*. Using this implementation, we have demonstrated TAAM-based structure factor and gradient calculations with runtimes comparable to existing direct summation IAM approaches.

By integration of the *pyDiSCaMB* library into the *Phenix* framework, TAAM refinement can become widely available to the structural biology community, potentially improving model quality for a variety of structures.

## Supplementary Material

Table of runtimes. DOI: 10.1107/S1600576726000828/ui5040sup1.pdf

## Figures and Tables

**Figure 6:**
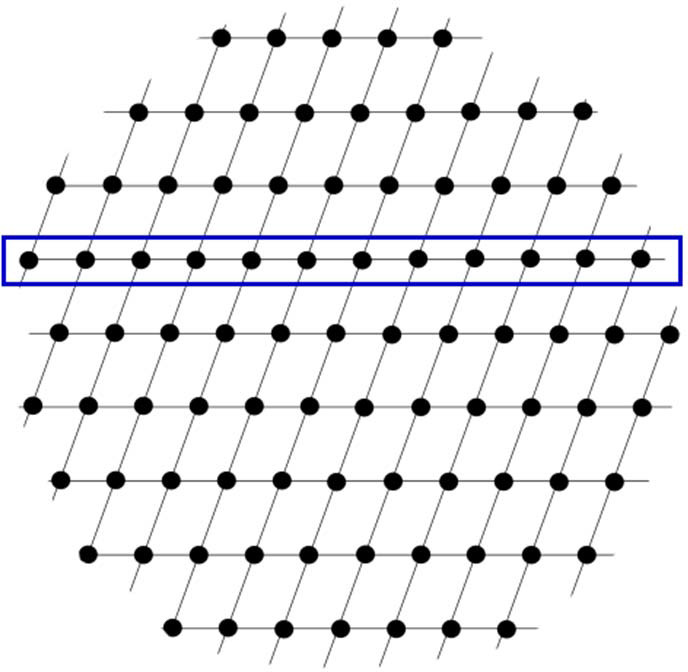
A 2D reciprocal-space representation of reflection points, with a row of equidistant reflections (blue box) highlighted.

## Appendix A Algorithm for speeding up structure factor calculations with direct summation

The algorithm focuses on speeding up calculations of exponential, sine and cosine functions, which appear in the atomic contribution to the structure factor:

The exponential function appears in the ‘temperature factor’  and the sine and cosine functions in the ‘phase factor’ . For equidistant reflections arranged along a line (Fig. 6 ▸), the two factors can be calculated efficiently without the need to calculate the exponent, sine and cosine at each point. For the phase factor, the value for the first reflection **h** on the line is

and for the next one at  it is

In general  for neighbouring points along the line. The multiplier *b* is calculated only once per line, and the calculation of subsequent phase factors involves only multiplication. Such an algorithm is used *e.g.* in diffuse scattering calculations (personal communication, T. Weber, 2009).

In the case of temperature factors *T* the procedure is slightly more complicated – the multipliers also have to be updated using a secondary multiplier. In the case of the isotropic displacement parameter *U*_iso_ this is

with

and in the case of the anisotropic displacement parameter tensor **U** it is

## Appendix B Availability and installation

The Git repository for *pyDiSCaMB* is available on GitHub at https://github.com/discamb-project/pyDiSCaMB under the MIT License. This includes the source code, a git submodule of *DiSCaMB* and the MATTS data bank, tests, scripts, and documentation in the form of example *Jupyter* notebooks. *DiSCaMB* is publicly available under the MIT License (https://github.com/discamb-project/DiSCaMB). The MATTS databank is publicly available for non-commercial use under a free licence (https://github.com/discamb-project/MATTS).

Scripts used to produce data for the figures in this paper are available as follows. Fig. 2 ▸ was obtained using the branch measure-runtime. All other figures were produced with the branch paper2025. Fig. 3 ▸ was generated using the script scripts/measure_fcalc_gradients_runtime.py together with pre-processed models provided in scripts/example/. Fig. 4 ▸ was generated using doc/electrostatic_potential_map.py and doc/electron_density_map.py. The X-ray deformation maps in Fig. 5 ▸ were computed using the notebook doc/deformation_map.ipynb. To calculate the deformation maps for electron diffraction in Fig. 5 ▸, the parameter xrs.scattering_type_registry(table="X-ray") can be changed to xrs.scattering_type_registry(table="electron") in the notebook.

Integration with *cctbx* and/or *Phenix* requires installation directly from the source code, as pre-built packages are not yet available. Installing *pyDiSCaMB* requires a C++ compiler and either *cctbx* (Version 2025.8) or *Phenix* (Version 2.0-5848 or later). Installation with *cctbx* additionally requires that *conda* is already installed. To install *conda*, the user must download the *Miniconda* or *Anaconda* installer for their operating system from https://www.anaconda.com/download and then follow the platform-specific instructions.

Installation in *cctbx* using *conda* (*cctbx* is installed in the first *conda* command) is as follows:[Chem scheme1]

Installation in *Phenix* using *pip* (requires *Phenix* Version 2.0-5848 or later; MacOS also needs *conda* to be installed) is as follows:[Chem scheme2]

With *pyDiSCaMB* installed, *cctbx* can compute TAAM structure factors and gradients by supplying algorithm="taam":[Chem scheme3]

This integration relies on changes made in Version 2025.8 of *cctbx*. A more complete reference of the interface is available in doc/cctbx_integration.ipynb.

## References

[bb1] Abrahams, D. & Grosse-Kunstleve, R. W. (2003). *C/C++ Users J.* **21**(7), 29–36.

[bb2] Adams, P. D., Afonine, P. V., Bunkóczi, G., Chen, V. B., Davis, I. W., Echols, N., Headd, J. J., Hung, L.-W., Kapral, G. J., Grosse-Kunstleve, R. W., McCoy, A. J., Moriarty, N. W., Oeffner, R., Read, R. J., Richardson, D. C., Richardson, J. S., Terwilliger, T. C. & Zwart, P. H. (2010). *Acta Cryst.* D**66**, 213–221. 10.1107/S0907444909052925 PMC2815670 20124702

[bb3] Adams, P. D., Grosse-Kunstleve, R. W., Hung, L.-W., Ioerger, T. R., McCoy, A. J., Moriarty, N. W., Read, R. J., Sacchettini, J. C., Sauter, N. K. & Terwilliger, T. C. (2002). *Acta Cryst.* D**58**, 1948–1954. 10.1107/s0907444902016657 12393927

[bb4] Adams, P. D., Pannu, N. S., Read, R. J. & Brünger, A. T. (1997). *Proc. Natl Acad. Sci. USA* **94**, 5018–5023. 10.1073/pnas.94.10.5018 PMC24623 9144182

[bb5] Afonine, P. V., Grosse-Kunstleve, R. W., Adams, P. D., Lunin, V. Y. & Urzhumtsev, A. (2007). *Acta Cryst.* D**63**, 1194–1197. 10.1107/S0907444907046148 PMC2808317 18007035

[bb6] Afonine, P. V., Lunin, V. Y., Muzet, N. & Urzhumtsev, A. (2004). *Acta Cryst.* D**60**, 260–274. 10.1107/S0907444903026209 14747702

[bb7] Afonine, P. V. & Urzhumtsev, A. (2004). *Acta Cryst.* A**60**, 19–32. 10.1107/s0108767303022062 14691324

[bb8] Berman, H. M., Westbrook, J., Feng, Z., Gilliland, G., Bhat, T. N., Weissig, H., Shindyalov, I. N. & Bourne, P. E. (2000). *Nucleic Acids Res.* **28**, 235–242. 10.1093/nar/28.1.235 PMC102472 10592235

[bb9] Bricogne, G. & Irwin, J. (1996). *Macromolecular Refinement: Proceedings of the CCP4 Study Weekend*, edited by E. Dodson, M. Moore, A. Ralph & S. Bailey, pp. 85–92. Warrington: Daresbury Laboratory.

[bb10] Capelli, S. C., Bürgi, H.-B., Dittrich, B., Grabowsky, S. & Jayatilaka, D. (2014). *IUCrJ* **1**, 361–379. 10.1107/S2052252514014845 PMC4174878 25295177

[bb11] Chen, V. B., Arendall, W. B., Headd, J. J., Keedy, D. A., Immormino, R. M., Kapral, G. J., Murray, L. W., Richardson, J. S. & Richardson, D. C. (2010). *Acta Cryst.* D**66**, 12–21. 10.1107/S0907444909042073 PMC2803126 20057044

[bb12] Chodkiewicz, M. L., Migacz, S., Rudnicki, W., Makal, A., Kalinowski, J. A., Moriarty, N. W., Grosse-Kunstleve, R. W., Afonine, P. V., Adams, P. D. & Dominiak, P. M. (2018). *J. Appl. Cryst.* **51**, 193–199. 10.1107/S1600576717015825 PMC5822993 29507550

[bb13] Clementi, E. & Roetti, C. (1974). *At. Data Nucl. Data Tables* **14**, 177–478.

[bb14] Cowley, J. M., Peng, L. M., Ren, G., Dudarev, S. L. & Whelan, M. J. (2006). *International Tables for Crystallography*, Vol. C, ch. 4.3, pp. 259–429. International Union of Crystallography.

[bb15] Davis, I. W., Murray, L. W., Richardson, J. S. & Richardson, D. C. (2004). *Nucleic Acids Res.* **32**, W615–W619. 10.1093/nar/gkh398 PMC441536 15215462

[bb17] Delano, W. L. (2002). *CCP4 Newsl. Protein Crystallogr.* **40**, 44–53.

[bb18] Dittrich, B., Hübschle, C. B., Pröpper, K., Dietrich, F., Stolper, T. & Holstein, J. J. (2013). *Acta Cryst.* B**69**, 91–104. 10.1107/S2052519213002285 23719696

[bb19] Dittrich, B., Weber, M., Kalinowski, R., Grabowsky, S., Hübschle, C. B. & Luger, P. (2009). *Acta Cryst.* B**65**, 749–756. 10.1107/S0108768109046060 19923703

[bb20] Dolomanov, O. V., Bourhis, L. J., Gildea, R. J., Howard, J. A. K. & Puschmann, H. (2009). *J. Appl. Cryst.* **42**, 339–341.

[bb21] Domagała, S., Fournier, B., Liebschner, D., Guillot, B. & Jelsch, C. (2012). *Acta Cryst.* A**68**, 337–351. 10.1107/S0108767312008197 22514066

[bb22] Dominiak, P., Kumar, A., Suresh, A., Lanza, A., Wojciechowski, J., Trzybiński, D., Brazda, P. & Palatinus, L. (2025). *Experimental Charge Density of Organic Nanocrystals Revealed by 3D Electron Diffraction*, https://doi.org/10.21203/rs.3.rs-7433721/v1.

[bb23] Dominiak, P. M., Volkov, A., Li, X., Messerschmidt, M. & Coppens, P. (2007). *J. Chem. Theory Comput.* **3**, 232–247. 10.1021/ct6001994 26627168

[bb24] Fillion-Robin, J.-C., McCormick, M., Padron, O., Smolens, M., Grauer, M. & Sarahan, M. (2018). *scikit-build*, https://github.com/jcfr/scipy_2018_scikit-build_talk.

[bb26] Grosse-Kunstleve, R. W., Sauter, N. K. & Adams, P. D. (2004). *IUCr CompComm. Newsl.* **3**, 21–31.

[bb27] Grosse-Kunstleve, R. W., Sauter, N. K., Moriarty, N. W. & Adams, P. D. (2002). *J. Appl. Cryst.* **35**, 126–136.

[bb28] Gruza, B., Chodkiewicz, M. L., Krzeszczakowska, J. & Dominiak, P. M. (2020). *Acta Cryst.* A**76**, 92–109. 10.1107/S2053273319015304 PMC8127334 31908353

[bb29] Guillot, B., Jelsch, C., Podjarny, A. & Lecomte, C. (2008). *Acta Cryst.* D**64**, 567–588. 10.1107/S0907444908006082 18453693

[bb30] Guillot, B., Viry, L., Guillot, R., Lecomte, C. & Jelsch, C. (2001). *J. Appl. Cryst.* **34**, 214–223.

[bb31] Hansen, N. K. & Coppens, P. (1978). *Acta Cryst.* A**34**, 909–921.

[bb32] Hirano, Y., Takeda, K. & Miki, K. (2016). *Nature* **534**, 281–284. 10.1038/nature18001 27279229

[bb35] Hofer, G., Wieser, S., Bogdos, M. K., Gattinger, P., Nakamura, R., Ebisawa, M., Mäkelä, M., Papadopoulos, N., Valenta, R. & Keller, W. (2019). *Allergy* **74**, 1009–1013. 10.1111/all.13696 PMC6563530 30515829

[bb36] Housset, D., Benabicha, F., Pichon-Pesme, V., Jelsch, C., Maierhofer, A., David, S., Fontecilla-Camps, J. C. & Lecomte, C. (2000). *Acta Cryst.* D**56**, 151–160. 10.1107/s0907444999014948 10666594

[bb37] Hunter, J. D. (2007). *Comput. Sci. Eng.* **9**, 90–95.

[bb38] Jakob, W., Rhinelander, J. & Moldovan, D. (2017). *pybind11*, https://github.com/pybind/pybind11_json/.

[bb39] Jayatilaka, D. & Dittrich, B. (2008). *Acta Cryst.* A**64**, 383–393. 10.1107/S0108767308005709 18421128

[bb40] Jelsch, C., Guillot, B., Lagoutte, A. & Lecomte, C. (2005). *J. Appl. Cryst.* **38**, 38–54.

[bb41] Jelsch, C., Pichon-Pesme, V., Lecomte, C. & Aubry, A. (1998). *Acta Cryst.* D**54**, 1306–1318. 10.1107/s0907444998004466 10089507

[bb42] Jelsch, C., Teeter, M. M., Lamzin, V., Pichon-Pesme, V., Blessing, R. H. & Lecomte, C. (2000). *Proc. Natl Acad. Sci. USA* **97**, 3171–3176. 10.1073/pnas.97.7.3171 PMC16211 10737790

[bb43] Jha, K. K., Gruza, B., Chodkiewicz, M. L., Jelsch, C. & Dominiak, P. M. (2021). *J. Appl. Cryst.* **54**, 1234–1243.

[bb44] Jha, K. K., Gruza, B., Kumar, P., Chodkiewicz, M. L. & Dominiak, P. M. (2020). *Acta Cryst.* B**76**, 296–306. 10.1107/S2052520620002917 32831250

[bb45] Jha, K. K., Gruza, B., Sypko, A., Kumar, P., Chodkiewicz, M. L. & Dominiak, P. M. (2022). *J. Chem. Inf. Model.* **62**, 3752–3765. 10.1021/acs.jcim.2c00144 PMC9400107 35943747

[bb47] Kohler, V., Goessweiner-Mohr, N., Aufschnaiter, A., Fercher, C., Probst, I., Pavkov-Keller, T., Hunger, K., Wolinski, H., Büttner, S., Grohmann, E. & Keller, W. (2018). *Nucleic Acids Res.* **46**, 9201–9219. 10.1093/nar/gky671 PMC6158623 30060171

[bb48] Krekel, H., Oliveira, B., Pfannschmidt, R., Bruynooghe, F., Laugher, B. & Bruhin, F. (2004). *pytest.* Version 8.4. https://github.com/pytest-dev/pytest/.

[bb49] Kulik, M., Chodkiewicz, M. L. & Dominiak, P. M. (2022). *Acta Cryst.* D**78**, 1010–1020. 10.1107/S2059798322005836 PMC9344478 35916225

[bb50] Kulik, M. & Dominiak, P. M. (2022). *Comput. Struct. Biotechnol. J.* **20**, 6237–6243. 10.1016/j.csbj.2022.10.018 PMC9676208 36420158

[bb51] Kulik, M. & Dominiak, P. M. (2025). *IUCrJ* **12**, 616–632. 10.1107/S2052252525008383 PMC12573929 41104950

[bb52] Kumar, P., Gruza, B., Bojarowski, S. A. & Dominiak, P. M. (2019). *Acta Cryst.* A**75**, 398–408. 10.1107/S2053273319000482 30821272

[bb53] Liebschner, D., Afonine, P. V., Baker, M. L., Bunkóczi, G., Chen, V. B., Croll, T. I., Hintze, B., Hung, L.-W., Jain, S., McCoy, A. J., Moriarty, N. W., Oeffner, R. D., Poon, B. K., Prisant, M. G., Read, R. J., Richardson, J. S., Richardson, D. C., Sammito, M. D., Sobolev, O. V., Stockwell, D. H., Terwilliger, T. C., Urzhumtsev, A. G., Videau, L. L., Williams, C. J. & Adams, P. D. (2019). *Acta Cryst.* D**75**, 861–877. 10.1107/S2059798319011471 PMC6778852 31588918

[bb54] Lunin, V. Yu. & Urzhumtsev, A. G. (1984). *Acta Cryst.* A**40**, 269–277.

[bb73] Lyskov, S. (2016). *PyRosetta-4*, https://www.pyrosetta.org/.

[bb55] Marques, M. A., Purdy, M. D. & Yeager, M. (2019). *Curr. Opin. Struct. Biol.* **58**, 214–223. 10.1016/j.sbi.2019.04.006 PMC6778505 31400843

[bb56] Maslen, E. N., Fox, A. G. & O’Keefe, M. A. (1992). *International Tables for X-ray Crystallography*, Vol. C, ch. 6.1.1. pp. 476–511. Dordrecht: Kluwer Academic Publishers.

[bb57] Maslen, E. N., Fox, A. G. & O’Keefe, M. A. (2006). *International Tables for Crystallography*, Vol. C, pp. 554–595. International Union of Crystallography.

[bb58] Massey, H. S. W. (1956). *Atoms II/Atome II*, edited by F. Hund, P. Gombás & H. S. W. Massey, pp. 232–306. Berlin, Heidelberg: Springer.

[bb85] Moriarty, N. W. (2015). *Comput. Crystallogr. Newsl.* **6**, 26.

[bb59] Murshudov, G. N., Vagin, A. A. & Dodson, E. J. (1997). *Acta Cryst.* D**53**, 240–255. 10.1107/S0907444996012255 15299926

[bb60] Olech, B., Brázda, P., Palatinus, L. & Dominiak, P. M. (2024). *IUCrJ* **11**, 309–324. 10.1107/S2052252524001763 PMC11067749 38512772

[bb61] Pacoste, L., Ignat’ev, V. M., Dominiak, P. M. & Zou, X. (2024). *IUCrJ* **11**, 878–890. 10.1107/S2052252524006730 PMC11364031 39146197

[bb62] Pannu, N. S., Murshudov, G. N., Dodson, E. J. & Read, R. J. (1998). *Acta Cryst.* D**54**, 1285–1294. 10.1107/s0907444998004119 10089505

[bb63] Pannu, N. S. & Read, R. J. (1996). *Acta Cryst.* A**52**, 659–668.

[bb64] Petříček, V., Dušek, M. & Palatinus, L. (2014). *Z. Kristallogr. Cryst. Mater.* **229**, 345–352.

[bb65] Petříček, V., Palatinus, L., Plášil, J. & Dušek, M. (2023). *Z. Kristallogr. Cryst. Mater.* **238**, 271–282.

[bb66] Read, R. J. (1986). *Acta Cryst.* A**42**, 140–149.

[bb67] Read, R. J. (1990). *Acta Cryst.* A**46**, 900–912.

[bb68] Rybicka, P. M., Kulik, M., Chodkiewicz, M. L. & Dominiak, P. M. (2022). *J. Chem. Inf. Model.* **62**, 3766–3783. 10.1021/acs.jcim.2c00145 PMC9400106 35943739

[bb69] Sayre, D. (1951). *Acta Cryst.* **4**, 362–367.

[bb70] Schmidt, A., Teeter, M., Weckert, E. & Lamzin, V. S. (2011). *Acta Cryst.* F**67**, 424–428. 10.1107/S1744309110052607 PMC3080141 21505232

[bb72] Schrödinger (2015). *The pyMOL Molecular Graphics System*. Version 1.8. https://www.pymol.org/.

[bb74] Sharma, D., De Falco, L., Padavattan, S., Rao, C., Geifman-Shochat, S., Liu, C.-F. & Davey, C. A. (2019). *Nat. Commun.* **10**, 5751. 10.1038/s41467-019-13641-0 PMC6917767 31848352

[bb76] Tanaka, I., Nishinomiya, R., Goto, R., Shimazaki, S. & Chatake, T. (2021). *Acta Cryst.* D**77**, 288–292. 10.1107/S2059798321000346 PMC7919404 33645532

[bb77] Ten Eyck, L. F. (1977). *Acta Cryst.* A**33**, 486–492.

[bb78] Tickle, I. J., Laskowski, R. A. & Moss, D. S. (1998). *Acta Cryst.* D**54**, 547–557. 10.1107/s0907444997013875 9761849

[bb79] Volkov, A., Macchi, P., Farrugia, L., Gatti, C., Mallinson, P., Richter, T. & Koritsanszky, T. (2024). *XD2024*, https://www.chem.gla.ac.uk/~louis/xd-home/.

[bb80] Waasmaier, D. & Kirfel, A. (1995). *Acta Cryst.* A**51**, 416–431.

[bb82] Williams, C. J., Headd, J. J., Moriarty, N. W., Prisant, M. G., Videau, L. L., Deis, L. N., Verma, V., Keedy, D. A., Hintze, B. J., Chen, V. B., Jain, S., Lewis, S. M., Arendall, W. B. III, Snoeyink, J., Adams, P. D., Lovell, S. C., Richardson, J. S. & Richardson, D. C. (2018). *Protein Sci.* **27**, 293–315. 10.1002/pro.3330 PMC5734394 29067766

[bb83] Yonekura, K., Kato, K., Ogasawara, M., Tomita, M. & Toyoshima, C. (2015). *Proc. Natl Acad. Sci. USA* **112**, 3368–3373. 10.1073/pnas.1500724112 PMC4372003 25730881

