# Supplementary material for: pyDiSCaMB: enabling the use of multipolar scattering factors in Phenix
Source: J Appl Crystallogr. 2026 Mar 20;59(Pt 2):662–72. doi: 10.1107/S1600576726000828 (PMC13060618; doi:10.1107/S1600576726000828)
Supplement: Supplementary file 1 [file j-59-00662-sup1.pdf]

JOURNAL OF APPLIED CRYSTALLOGRAPHY

Volume 59 (2026)

Supporting information for article:

pyDiSCaMB: enabling the use of multipolar scattering factors in *Phenix*

Viljar J. Femoen, Laura Pacoste, Michał Leszek Chodkiewicz, Pavel V. Afonine, Billy K. Poon, Marta Kulik, Łukasz Golon, Nigel W. Moriarty, Paul D. Adams, Gerhard Hofer, Paulina Maria Dominiak, Dorothee Liebschner and Xiaodong Zou

Table S1 Mean runtime (n=5) and standard deviations of computing structure factors and target gradients for selected entries in the PDB.

PDB ID	Calculation method	Fcalc runtime		Target gradient runtime	
		mean	std	mean	std
3NiR	cctbx IAM (FFT)	0.081	0.002	0.267	0.001
7DER		0.0855	0.0008	0.254	0.002
6G1T		0.0236	0.0004	0.0646	0.0002
6GER		0.0548	0.0005	0.187	0.001
6iPU		0.250	0.002	0.716	0.001
3NiR	cctbx IAM	1.36	0.03	2.632	0.004
7DER		4.46	0.02	8.94	0.02
6G1T		0.87	0.01	1.720	0.007
6GER		6.333	0.006	12.53	0.02
6iPU		41.4	0.2	90.18	0.16
3NiR	DiSCaMB IAM	0.96	0.02	5.34	0.02
7DER		3.25	0.03	19.952	0.010
6G1T		5.72	0.02	6.310	0.010
6GER		5.12	0.03	27.97	0.02
6iPU		49.3	0.1	255.7	0.2
3NiR	DiSCaMB TAAM	2.86	0.03	3.933	0.010
7DER		8.28	0.02	11.38	0.02
6G1T		5.31	0.01	6.06	0.01
6GER		12.10	0.04	16.73	0.03
6iPU		125.9	0.2	157.3	0.4
3NiR	DiSCaMB TAAM (MT)	0.345	0.009	0.603	0.007
7DER		0.88	0.01	1.634	0.005
6G1T		0.76	0.02	1.045	0.009
6GER		3.63	0.06	5.38	0.04
6iPU		50.2	0.1	67.8	0.1
